# Choices Today, Behaviours Tomorrow: Longitudinal Associations Between Childhood Risky Decision-making and Adolescent Conduct Disorder Behaviours – a Nationally Representative Prospective Cohort Study in the United Kingdom

**DOI:** 10.1192/bjo.2024.76

**Published:** 2024-08-01

**Authors:** Christy Cheung, Gemma Lewis, Glyn Lewis, Ramya Srinivasan

**Affiliations:** UCL Division of Psychiatry, London, United Kingdom

## Abstract

**Aims:**

Conduct disorder carries significant individual and societal repercussions. Despite heightened risk-taking and challenges in adapting to changing probabilities of choice outcomes being linked to maladaptive behaviours such as conduct disorder, no study to date has examined the association behind childhood decision-making and adolescent conduct disorder. This study seeks to address this gap by exploring the longitudinal association between these two variables. Understanding the mechanisms underlying conduct disorder could help with developing new preventive interventions.

**Methods:**

We used data from the Millennium Cohort Study, a nationally representative UK cohort; participants included those with complete data on exposure, outcome and confounding variables (n = 7,237). The exposure, childhood decision-making at 11 years was measured using the Cambridge Gambling Task risk-taking and risk-adjustment measures. The outcome, a binary measure of adolescent conduct disorder was created using items from the risky and antisocial behaviour interview sections at age 17. We used logistic regression to examine the association between childhood decision-making and adolescent conduct disorder and adjusted for relevant confounders.

**Results:**

The univariable model showed that at age 11, each 20-point increase in risk-taking score increased the odds of conduct disorder behaviour at age 17 by 32% (OR = 1.32, 95% CI 1.18–1.44, p < 0.0001). In the multivariable model, there was strong evidence that a 20-point increase in risk-taking at 11 years was associated with 18% higher odds of conduct disorder behaviour at 17 years (OR = 1.18, 95% CI 1.05–1.33, p = 0.005). There was no evidence that this association differed by sex. Risk adjustment at 11 years showed no association with conduct disorder behaviours at age 17 both in the univariable model (OR = 0.96, 95% CI 0.88–1.06, p = 0.440) and the multivariable model (OR = 0.96, 95% CI 0.88–1.06, p = 0.433).

**Conclusion:**

We found that risk-taking at 11 years was associated with conduct disorder behaviour at 17 years. If causal, our findings suggest that risk-taking might be a potential mechanism underlying adolescent conduct disorder behaviours. This may be useful in informing the design of preventive strategies, such as encouraging positive risk-taking in children and discouraging negative risk-taking behaviours.

